# Determination of the Three-Dimensional Rate of Cancer Cell Rotation in an Optically-Induced Electrokinetics Chip Using an Optical Flow Algorithm

**DOI:** 10.3390/mi9030118

**Published:** 2018-03-08

**Authors:** Yuliang Zhao, Dayu Jia, Xiaopeng Sha, Guanglie Zhang, Wen Jung Li

**Affiliations:** 1 School of Control Engineering, Northeastern University at Qinhuangdao, Qinhuangdao 066004, China; jiadayu.neu@gmail.com (D.J.); Shaxiaopeng@neuq.edu.cn (X.S.); 2 Department of Mechanical and Biomedical Engineering, City University of Hong Kong, Kowloon, Hong Kong 999077, China; 3 Shenzhen Academy of Robotics, Shenzhen 518000, China; guanglie.zhang@gmail.com

**Keywords:** cell self-rotation, single cell rotation, optically induced electrokinetics, optical flow algorithm

## Abstract

Our group has reported that Melan-A cells and lymphocytes undergo self-rotation in a homogeneous AC electric field, and found that the rotation velocity of these cells is a key indicator to characterize their physical properties. However, the determination of the rotation properties of a cell by human eyes is both gruesome and time consuming, and not always accurate. In this paper, a method is presented to more accurately determine the 3D cell rotation velocity and axis from a 2D image sequence captured by a single camera. Using the optical flow method, we obtained the 2D motion field data from the image sequence and back-project it onto a 3D sphere model, and then the rotation axis and velocity of the cell were calculated. After testing the algorithm on animated image sequences, experiments were also performed on image sequences of real rotating cells. All of these results indicate that this method is accurate, practical, and useful. Furthermore, the method presented there can also be used to determine the 3D rotation velocity of other types of spherical objects that are commonly used in microfluidic applications, such as beads and microparticles.

## 1. Introduction

Cell rotation is a basic function that researchers use to manipulate single cells for genetic and cellular property studies. For example, the rotation of cells to a proper position before injection of foreign DNAs into cells or biopsy of the intracellular structures, is a process that is commonly utilized in genetic studies and in stem cell research, as well as in clinical in vitro fertilization procedures [1]. Cell rotation is also a key technique required to perform studies on single-cell phenotypic heterogeneity and can reveal more information about the pathogenesis process than conventional bulk methods [2]. In addition, the differences in the cell rotation velocity between different types of cells could possibly be used as a biomarker for quick discrimination among cells with different morphological features, electrical properties, or functions [3,4].

During the last few decades, various techniques have been developed to achieve controlled cell rotation. The resulting rotation behaviors of cells in a rotating electric field have been reported [5,6,7]. Essentially, when a cell is exposed to an electric field, a dipole is induced. If the field is rotating with a sufficiently high frequency, the cell will experience torque and begin to rotate [8]. The main difference between these techniques is that the rotating electric field was either produced by dielectrophoresis (DEP) or by optically-induced electrokinetics (OEK). Microfluidic devices are also a versatile platform for cell rotation due to unique micro-flow phenomena. For instance, Zhang recently developed a micro-vortex chamber that allows living cells to be rotated solely by hydrodynamic forces [2] and Leung used a standard micropipette to generate a fluidic flow to rotate mouse embryo cells [1]. On the other hand, optical tweezers (also known as laser trapping) use a focused laser beam to manipulate microscopic objects and are also capable of rotating cells [9]. The rotation of magnetically-labeled cells in the presence of a magnetic field has also been reported [10].

### 1.1. Self-Rotation of a Single Cell in the OEK Chip

The phenomenon of self-induced rotation motion in cells in a homogeneous AC electric field was observed and reported recently. Specifically, two new phenomena were discovered by our group: the self-induced rotation motion of pigmented biological cells in a DEP force field [11] and the self-rotation of Melan-A pigmented cells and three types of leukemia cells induced by the OEK [3,4,12]. Hence, accurate determination of the cell rotation velocity is likely to become an important means to predict the dielectric property, charge distribution, and other physical properties of different types of cells. In this paper, we will present an automated method to quantify the cell rotation velocity.

According to the electromagnetic and hydrodynamic theories, the OEK rotation torque and the fluidic shear torque acting on a cell suspended in a fluid are calculated using Equations (1) and (2) [6]:

(1) Γ→DEP=4πr3⋅εm⋅Im[K(ω)]⋅Erms2⋅j^

(2) $${\vec{\Gamma }}_{f}=8\pi \eta Vr^{3}\cdot \hat{j}$$

where V is the cell rotation velocity, r is the cell radius, $\varepsilon_{m}$ is the permittivity of the media surrounding the cell, $E_{rms}^{2}$ is the root-mean-square value of the electric field $\vec{E}$, and $\hat{j}$ denotes the unit vector of the axis. η is the viscosity of the medium, ω is the applied angular frequency across the medium, and K(ω) is the Clausius-Mossotti (CM) factor, which is described by Equation (3):

(3) $$K(\omega )=\frac{\varepsilon_{p}^{*}-\varepsilon_{m}^{*}}{\varepsilon_{p}^{*}+2\varepsilon_{m}^{*}}$$

where $\varepsilon_{p}^{*}$ and $\varepsilon_{m}^{*}$ are the complex permittivity of the cell and the medium. $\varepsilon_{m}^{*}$ is defined by Equation (4):

(4) $$\varepsilon^{*}=\varepsilon -j\frac{\sigma }{\omega }$$

where ε and σ refer to the permittivity and conductivity, respectively. If the cell rotation velocity *V* is known, the equilibrium conditions can be represented by Equation (5):

(5) $${\vec{\Gamma }}_{DEP}+{\vec{\Gamma }}_{f}^{}=0$$

Substituting Equations (1)–(4) into Equation (5) yields an equation for ε and σ. The cell’s permittivity and conductivity can be calculated easily at the two different induced rotation velocities.

### 1.2. Video-Based Rotation Analysis

Determining the cell rotation velocity from a video or a time-lapsed series of microscope images is challenging. Most of the methods are asynchronous or are limited to a fixed rotation axis, so they are unable to manipulate a cell precisely or quantify a cell’s rotation velocity accurately.

**Figure 1:**
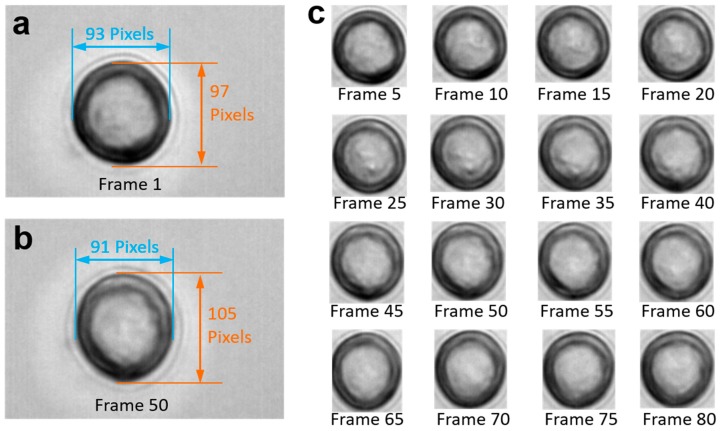
Video frames of rotating cells with cell image details and time-lapse image capture sequence. (**a**) Image of the original gesture of the cell. The length of the major axis is 97 pixels, while that of the minor one is 93 pixels; (**b**) after rotation, the major and minor axis lengths of the cell change to 105 and 91 pixels respectively; and (**c**) image sequence of the region of interest. The video was taken at 100 frames per second.

The video frames in Figure 1 show a cell rotating around an unknown axis. Note that the cell is transparent, and only some of the gray texture on the cell surface is visible. However, we cannot ascertain whether the gray texture is on the upper hemisphere or on the bottom hemisphere, or even whether it is an artifact resulting from optical interference between the camera and the projector. As stated by De Gasperisy, real-time motion detection of biological cells is a challenging issue because cells cannot be modeled as rigid bodies due to their translucent, refractive, and diffractive appearance [13]. All these three types of disturbances can result in distortion and calculation errors.

Other researchers used a particle image velocimetry (μ-PIV) system seeded with fluorescent and sufficiently-small tracer particles to calculate the cell rotation velocity [2]. Obviously, it is not suitable for our experimental conditions. The accuracy of this PIV method is often affected by the stagnant nature of the solution and the unfocused or occluded particles, which may generate incorrect velocity vectors. The most challenging aspect of a μ-PIV measurement is the non-uniform distribution and the asynchronous rotation. Another computer-based machine vision algorithm and hardware implementation have been presented by De Gasperis to measured cell rotation motion and analyzed the electrorotation (ROT) spectra [13]. After the image of a new frame centered on the cell was grabbed, the cell’s image was transformed from the Cartesian space into polar coordinates. The authors used an image segmentation and contour extraction method based on the snake model for the cell image in the electrorotation chip video they presented. The method also utilized the least-squares ellipse-fitting algorithm to describe the rotation characteristics of cells in a rotating electric field [14,15]. Since the rotation axis of the cell is vertical to the image plane, the cell’s rotation angle can be indicated by the horizontal deflection angle of the major axis of an ellipse. The difference in the horizontal deflection angles between two sequential video frames is stored and used to calculate the cell rotation speed.

In this paper, we present an algorithm for precisely tracking the rotation and calculating the rotation velocity of a cell. We have also shown that this algorithm can be further applied to determine a cancer cell’s rotation velocity in a homogeneous AC electric field. This algorithm starts by obtaining the position of the target cell in the 2D images. In contrast to existing approaches that only calculate the planar (yaw-axis) rotation of an object from a 2D image [1,13,14,16], this algorithm first calculates the velocity of each point on the 2D plane and then projects the results to the surface of a 3D cell model. The 3D cell model can rotate about any axis. After a geometric operation, the rotation axis and velocity are calculated. The method discussed here provides a new way to determine the rotation velocity of single cells repaidly, and can also be applied to ascertaion the rotation velocity of other spherical objects.

## 2. Methods

**Figure 2:**
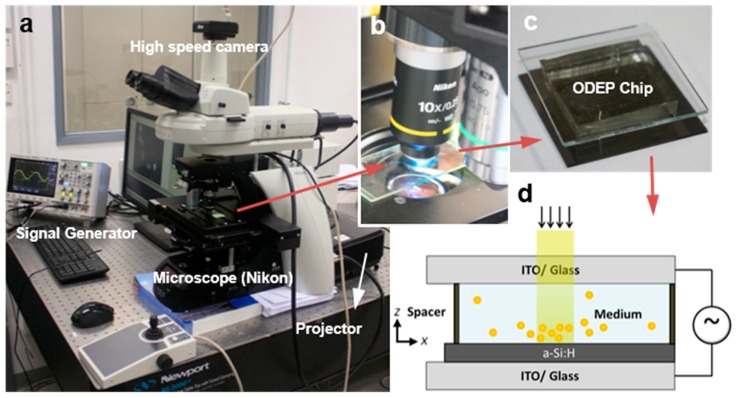
Experimental setup of an OEK system. (**a**) The complete OEK system; (**b**) the light from a digital projector passing through the chips. The cell rotation images are obtained by a high-speed CCD placed to the top of the microscope; and (**c**) the structure and (**d**) the working principle of the OEK chip.

The OEK technique allows for high-resolution patterning of virtual electric contacts on a photoconductive surface for manipulating single particles [17] and it can solve many problems associated with the manipulation of cells or micro-particles by leveraging electrokinetic forces [18,19]. Consisting of a smooth photoconductive layer between two conductive chips, a microscope station, a power supply, and a projection light source, an OEK system can be built easily and cheaply. The cell rotation video was captured by using the OEK system shown in Figure 2. The details about the cell rotation experiments, including the preparation and fabrication of the experiment setup, have already been provided in our previous publications [3,11,12]. Therefore, this paper will mainly discuss the algorithm for calculating the cell rotation velocity.

### 2.1. 3D Motion Recovery Using a Micro-Vision System

Traditional algorithms that are based on binocular vision are not applicable to this situation. This is because most micro-vision systems are monocular due to the narrow field of view and the short depth of focus of the microscope. Our experimental system also uses a single camera; which renders the depth of field information totally lost. Another common approach to 3D motion recovery is to choose an initial reference template and then match the images with this reference template. This method is widely used for recognition tracking and motion recovery of human facial and head gestures [20,21]. As the texture and the rotation axis are difficult to predict and there is no obviously-preferred direction for the cells, the template matching method is not feasible. More generally, feature extraction is the basis for image processing and computer vision. However, none of the traditional surface features, such as edges, corners, blobs, and ridges can be referenced. The block-matching method was developed by our group to track and calculate the self-rotation velocity of cells about a fixed axis [17,22]. This 2D method uses a mask to match cells in each frame. The resulting correlation coefficient between the mask and the frame reflects the similarity between them. The rotation velocity can be calculated through analyzing the periodical changes of the correlation coefficients. Thus, it can only calculate the average rotation velocity by identifying the time one round of cell rotation takes. After comparing various algorithms, we selected an optical flow method to obtain the velocity data. Since the optical flow method performs the calculation based on two adjacent image frames it can determine the instantaneous rotation velocity and axis, which are not attainable through block matching.

### 2.2. Algorithm for Calculating the Cell Rotation Velocity

**Figure 3:**
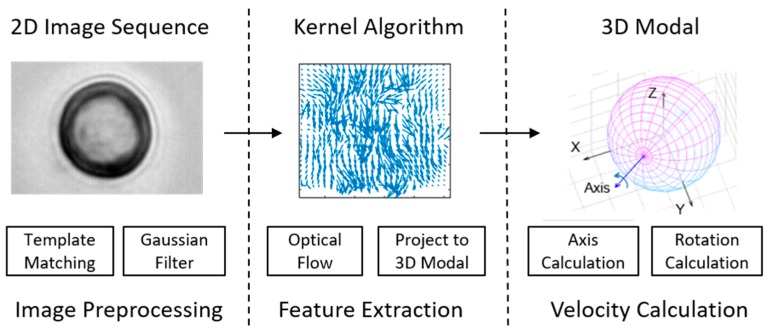
A flow diagram of the algorithm architecture.

After a video or an image sequence of the rotating cells is acquired by the digital camera, an algorithm based on the optical flow method is used to estimate the rotation. The algorithm can be divided into four main functions: cell recognition, image preprocessing, velocity feature extraction, and velocity feature recognition. The flow diagram for this algorithm is shown in Figure 3.

#### 2.2.1. Cell Recognition

The process starts with cell recognition via a template matching algorithm that identifies the region of interest in the image sequence. The correlation method is widely used for template matching and automated determination of any translational motion:

(6) $$R(i,j)=\frac{\sum_{m=1}^{M}\sum_{n=1}^{M}\left[S^{i,j}(n,m)\times T(n,m)\right]}{\sqrt{\sum_{m=1}^{M}\sum_{n=1}^{M}{\left[S^{i,j}(n,m)\right]}^{2}}\sqrt{\sum_{m=1}^{M}\sum_{n=1}^{M}{\left[T(n,m)\right]}^{2}}}$$

where T(n,m) and $S^{i,j}(n,m)$ are the average intensities of the template and local image window, respectively. The sequential similarity detection algorithms (SSDAs) [23,24] were adopted for the cell matching and cellular translation determinations. Proposed by Barnea and Silverman, SSDAs are more efficient than the traditional cross-correlation method and have been widely used for 40 years. The basic idea of this algorithm is that if the accumulated error in the computation of similarity is greater than a prescribed threshold, the computation is terminated. Thereby, it significantly reduces the computation time and enhances the overall matching speed.

When the search template *T* moves onto an image *S*, the windowing pairs $S^{i,j}(n^{k},m^{k})-S(i,j)$ and T(n,m)−T are compared in a random order, where $S^{i,j}$ is the sub-image covered by the template and i and j (1≤i,j≤N−M−1) are the coordinates of the top left point. Normalized measures for evaluating the error between the windowing pairs are defined as follows:

(7) $$\varepsilon (i,j,n_{k},m_{k})=\left|\left(S^{i,j}(n_{k},m_{k})-S(i,j))-(T(n,m)-T\right)\right|$$

(8) $$S(i,j)=\frac{1}{N^{2}}\sum_{n=1}^{N}\sum_{m=1}^{N}s^{i,j}(n,m)$$

(9) $$T=\frac{1}{N^{2}}\sum_{n=1}^{N}\sum_{m=1}^{N}t(n,m)$$

In this step, the cell’s 2D translation trajectory and the velocity are obtained along with the correlation during the matching process.

#### 2.2.2. Image Preprocessing

The main purpose of image preprocessing is denoising because all the subsequent calculations are based on the images obtained from this step. Possible sources of image noise include photon noise, thermal noise, readout noise, and quantization noise. These sources of noise are embodied as interferometric fringes, variations in brightness, fuzzy features, and Gaussian white-noise in a sequence of images shown in Figure 1. The optical flow method is built on the assumption that the brightness is constant for a particular point in the pattern and the image is smooth almost everywhere. Brightness indicates the overall whiteness or darkness of the image. Currently, no standard formula is available for calculating brightness [25]. In this study, the brightness of our grayscale images was measured by its mean grayscale value. We first calculated the total mean grayscale value of the image sequence and then adjusted the mean grayscale value of each image to this value.

With a proper low-pass filter, the random noise arising from the projector, the CCD or other parts of the image acquisition system can be removed to smoothed image. Gaussian filtering is well accepted as an optimal option for both smoothing and denoising, so we adopted a 2D isotropic Gaussian kernel to perform it in two separate orthogonal directions.

#### 2.2.3. Extracting Velocity Features

Optical flow is the distribution of apparent velocities resulting from the movement of brightness patterns in images. Its goal is to compute an approximation of the 2D motion field and a projection of the 3D velocities of surface points onto the imaging surface from spatiotemporal patterns of the image intensity [26,27]. By providing an accurate and dense approximation of the 2D motion field, the optical flow method makes it possible to measure the rotation velocity of the cell. Many methods for computing the optical flow have been developed since it was first proposed by Horn and Schunck (HS) [26]. The majority of the current methods strongly resemble the original HS formulation. Indeed, the typical formulation has changed little since the original publication. The “classical” flow formulations perform surprisingly well when combined with modern optimization and implementation techniques [28]. Barron and Fleet reported the results of a number of regularly cited optical flow techniques, including instances of differential, matching, energy-based, and phase-based methods [27]. Moreover, McCane, et al. provided a preliminary quantitative evaluation of seven optical flow algorithms using synthetic and real sequences. By comparing the performance of each algorithm on the most complex synthetic test scenarios, the HS method outperforms the other five methods [29].

The HS method was adopted to obtain the 2D optical flow field. As noted above, the HS method is mainly based on two assumptions. First, the brightness of a particular point in the pattern is constant, which means that the objects in the image should keep the same intensity value while moving, at least for a short period of time. This feature can be expressed as follows: ∀(x,y)∈Ω ∀t∈[0,T]:

(10) I(x,y,t)=I(x+dx,y+dy,t+dt)

Thus, the optical flow constraint equation (OFCE) is obtained by using a Taylor expansion and dropping the high-order nonlinear terms. The OFCE is expressed as follows:

(11) $$I_{x}u+I_{y}v+I_{t}=0$$

where (u,v) represents the horizontal and the vertical components of the optical flow field vectors (dx/dt,dy/dt) and $\left(I_{x},I_{y},I_{z}\right)$ represent the derivatives of the image intensities at coordinates (x,y,t).

The second assumption is that neighboring points on the objects have similar velocities and the velocity field of the brightness patterns in the image varies smoothly almost everywhere [26,27]. One way to express this additional constraint is to minimize the square of the magnitude of the gradient of the optical flow velocity:

(12) $$E_{s}=\iint \left(u_{x}^{2}+u_{y}^{2}+v_{x}^{2}+v_{y}^{2}\right)dxdy$$

(13) $$\min\iint \left[\lambda \left(u_{x}^{2}+u_{y}^{2}+v_{x}^{2}+v_{y}^{2}\right)+{\left(I_{x}u+I_{y}v+I_{t}\right)}^{2}\right]dxdy$$

**Figure 4:**
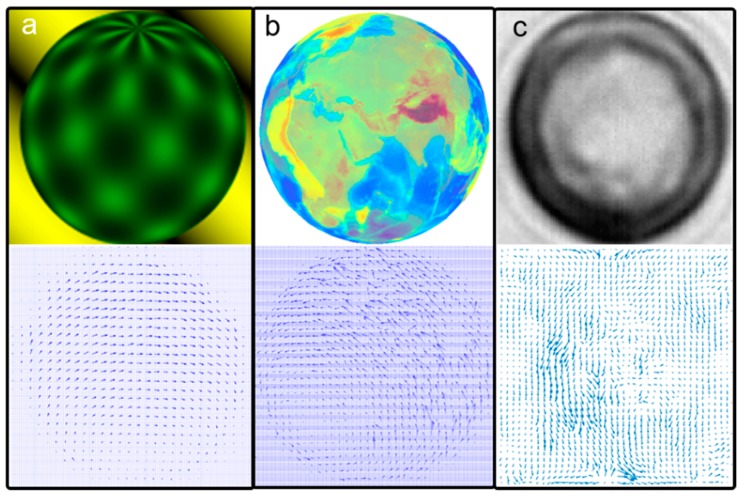
Image templates and their optical flow results. (**a**) Baker’s test template (126 × 126 pixels); (**b**) Earth globe test sequence (207 × 207 pixels); and (**c**) cell rotation sequence (101 × 101 pixels).

By using multivariate variations, we can find the solution (*u*, *v*) that minimizes the evaluation of Equation (13). This coupled system is symmetric with respect to the two components of the velocity u and v. HS solves these two equations simultaneously using block Gauss-Seidel relaxation to capture the coupling effect between them, which is expressed as:

(14) $$\begin{array}{c} u^{<n+1>}={\bar{u}}^{<n>}-I_{x}\left(\frac{I_{x}\bar{u}+I_{y}\bar{v}+I_{t}}{\alpha +I_{x}^{2}+I_{y}^{2}}\right)\\ v^{<n+1>}={\bar{v}}^{<n>}-I_{y}\left(\frac{I_{x}\bar{u}+I_{y}\bar{v}+I_{t}}{\alpha +I_{x}^{2}+I_{y}^{2}}\right) \end{array}$$

where $\left(\bar{u},\bar{v}\right)$ represents an average of the neighboring points to (u,v). The images of optical flow are computed using the first-order differentials of $\left(I_{x},I_{y},I_{z}\right)$, which have been approximated with the neighboring points in the successive image. Figure 4 shows an optical flow obtained using the algorithm illustrated above.

#### 2.2.4. Extracting Velocity Features

**Figure 5:**
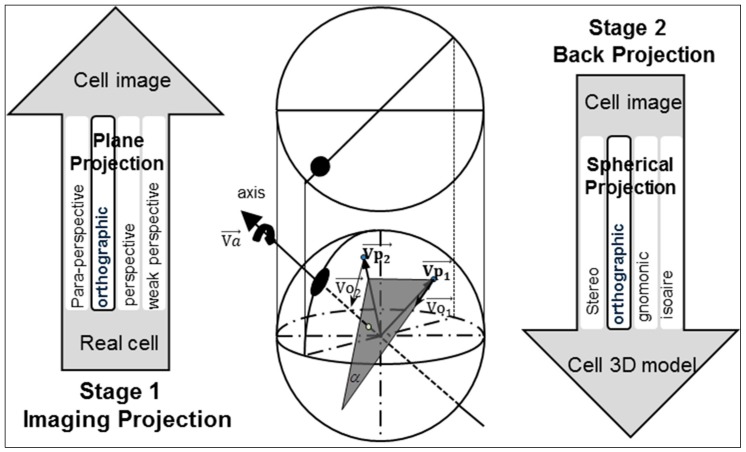
The image mapping process for the 2D and 3D models. In stage 1, the real cell projects its image onto the 2D plane, while in stage 2, the 2D image is back projected to a 3D model. $\vec{V_{p1}}$ and $\vec{V_{p2}}$ represent two vectors from the center of the sphere to the points on the sphere surface. $\vec{V_{o1}}$ and $\vec{V_{o2}}$ are their 3D optical flow vectors when the sphere rotates around the axis $\vec{V_{\alpha }}$.

The process involves two key projections: capturing the image of the real 3D cell using a 2D CCD camera and mapping of the 2D image data to the 3D cell model. Here, the first plane projection is referred to as imaging projection. The second spherical projection is referred to as back projection. To simplify the calculations, researchers have proposed many approximations for full-perspective projection [30]. Affine projection, weak perspective projection, para-perspective projection, and orthographic projection are the common models used for image projection. As reported in published literatures, orthographic projection has been widely used to preserve the 3D shape and motion recovery [31,32]. Orthographic projection is an approximation of perspective projection. It works when the object is close to the optical axis of the camera and its dimensions are relatively small compared to the distance from the camera. The dimension of the cell is approximately 10 µm, which is negligible compared to the distance from the cell to the camera. Thus, an orthographic projection was adopted for the imaging projection. The decision to employ orthographic projection was also motivated by the spherical projections [33,34] from a plane to the surface of a sphere. Among the four spherical projection methods, orthographic spherical projection is the simplest. This method will not magnify the error produced during the imaging projection process thanks to its orthographic projection approximation. It also compensates for the total systematic error because the orthographic projection is performed from a 2D image to a 3D model in the spherical projection steps, in a similar manner as an inverse transformation. Figure 5 shows the process for generating an orthographic projection from imaging projection and from back projection.

After a 2D velocity feature field is obtained using optical flow, the next key step is to use an orthographic projection to back project it onto the 3D cell model. This process transforms the 2D velocity field into 3D coordinates and then enables the identification of the rotation axis and calculation of the rotation velocity. The point (x,y) on the 2D image plane was transformed to the point (*x*, *y*, *z*) on the 3D sphere:

(15) $${\vec{V}}_{p}=[x,y,z]$$

We assume that the cell is in a shape of a perfect sphere. Hence, any point on the sphere will follow the constraint listed below:

(16) $$x^{2}+y^{2}+z^{2}=r^{2}$$

The radius r is obtained from the template matching step. By means of a back projection from the orthographic projection, the vector $\vec{V_{p}}$ can be calculated by Equation (16). In a similar way, the optical flow vector $\vec{V_{o}}$ of the point (x,y,z) on the 3D sphere can also be calculated:

(17) $$\vec{V_{o}}=\vec{U_{xy}}+\vec{V_{xy}}+\vec{Z_{xy}}$$

where $\vec{U_{xy}}$ and $\vec{V_{xy}}$ are the horizontal and vertical optical flow vector that were obtained from Equation (14). And the 3D optical flow vector $\vec{V_{o}}$ should be perpendicular to the vector $\vec{V_{p}}$ of the point (x,y,z) on the sphere:

(18) $$\vec{V_{o}}\times \vec{V_{p}}=0$$

Then, we can calculate the 3D rotation axis $\vec{V_{a}}$, because every 3D optical flow vector $\vec{V_{o}}$ should be perpendicular to the rotation axis $\vec{V_{\alpha }}$. Figure 5 illustrates the spatial relationship between these vectors. If a plane α meet the conditions (1) $\vec{V_{o1}}\in \alpha$ and (2) $\vec{V_{o2}}//\alpha$, then $\vec{V_{\alpha }}⊥\alpha$. The rotation axis can be calculated from optical flow vectors $\vec{V_{o1}}$ and $\vec{V_{o2}}$.

(19) $$\vec{V_{a}}=\left|\vec{V_{p}}\right|\times \frac{\vec{V_{o1}}\times \vec{V_{o2}}}{\left|\vec{V_{o1}}\cdot \vec{V_{o2}}\right|}$$

Based on $\vec{V_{a}}$ and $\vec{V_{p}}$, the distance *d* from a point on the sphere to the rotation axis can be calculated easily. Then, the rotation velocity *V* (unit: degree/frame) is:

(20) $$V=\frac{360^{\circ }\cdot \left|\vec{V_{o}}\right|}{2\pi d}$$

The rotation velocity between these two frames is determined using this optical flow method and can be applied easily to the entire image sequence. For an image sequence with *n* + 1 frames, we can obtain *n* instantaneous rotation velocities and axes. As the rotation amplitude between two adjacent frames is very small, the rotation axis can be treated as a fixed axis, which means that the change of the axis direction between two adjacent frames is neglected. However, the change of the axis direction during the whole cell rotation process can be described clearly using the *n* instantaneous rotation axes. After the rotation velocity and axis is calculated from the entire image sequence, it can be evaluated if there is a ground truth.

## 3. Results

**Figure 6:**
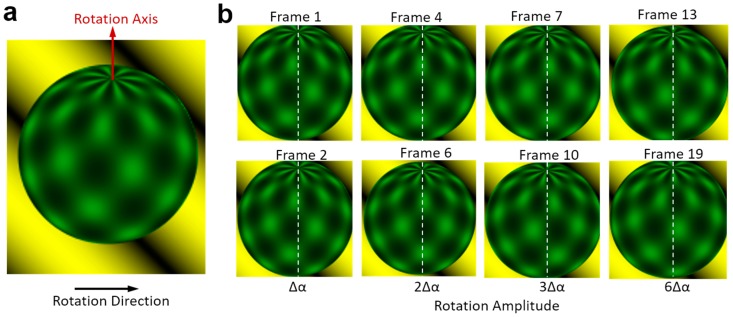
Baker’s test template. (**a**) An artificial ball rotates around an axis in the anti-clockwise direction looking in a top view; and (**b**) the image sequence of the region of interest. ∆α represents the angle between two adjacent images. The white dash line is the benchmarking for convenient comparison.

Firstly, the cell-rotation-rate algorithm was tested using animated image frames as shown in Figure 6. This database was published by Baker for optical flow algorithm evaluation [35]. It contains 46 image frames of a ball rotating with a fixed axis and amplitude. There is no temporal information about this image sequence. We calculated the rotation amplitude (unit: degree) of the ball between two images, which is also referred to as the rotation velocity (unit: degree/frame). The rotation amplitude calculated between two adjacent images is marked as ∆α, shown in Figure 6b. After calculating all the 45 rotation amplitudes between two adjacent images, we found ∆α = 2.12° ± 0.01°. This means that the ball rotates very uniformly and our method is accurate enough. To create more test sequences with different rotation amplitudes, we also calculated the rotation amplitude between the image*^i^* and the image*^(i+n)^*, where *i* indicates the frame number in the image sequence. *n* is the difference between the frame numbers, which indicates the number of times the rotation amplitude is ∆α. When the number of times of rotation is *n*, the rotation amplitude should be *n* times ∆α, which equals 2.12*n*°. We used this formula to describe the ground truth for comparing with the calculated angle using the algorithm shown in this paper.

**Figure 7:**
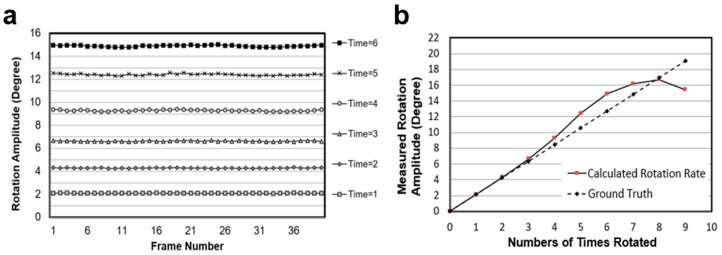
Evaluation of optical flow algorithm based Baker’s test template. (**a**) The rotation amplitude of the ball in different image sequences. In the legend, ‘*Time = n*’ represents how many times the rotation amplitude of the image sequence is that between two adjacent images, and *n* is the difference between the frame numbers; and (**b**) the comparison between the ground truth and the calculated rotation velocity on different testing sequences. The calculated rotation velocity is the average value of the same sequence.

Figure 7a shows the test results, which are more stable and convincible when these different rotation amplitude sequences are compared. Figure 7b shows the comparation of the calculated rotation velocities from different image sequences with the ground truth (2.12*n*°) defined above. These results demonstrate that the optical flow algorithm provides an accurate and stable method to calculate the rotation amplitude of a spherical object, especially in the range of 0–10°.

**Figure 8:**
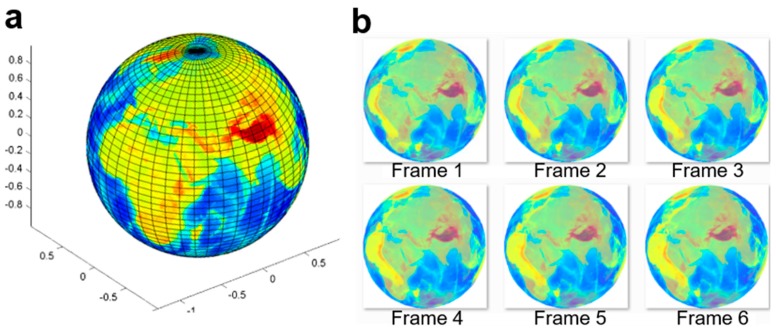
Animated Earth globe test template. (**a**) Original Earth globe image; and (**b**) sequentially-rotating images (2°/frame) of the Earth template (semi-transparent surface, 207 × 207 pixels).

To simulate the transparent cell member and the complicated optical disturbances, more animated image frames were generated to validate this algorithm. We created an animated database, where an Earth globe rotates with the ground truth and semi-transparent surface. In this template, the rotation velocity and the axis of the Earth globe are accurately controlled. Figure 8 shows the images of the time-lapse sequences of a rotating Earth globe, and Figure 4b shows the calculated optical flow between two adjacent frames.

**Figure 9:**
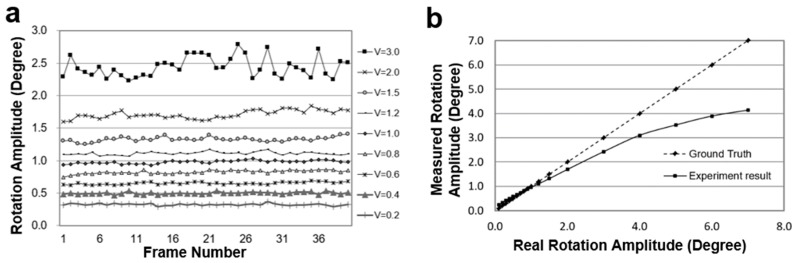
Test results on the animated rotating Earth globe. (**a**) The calculated rotation amplitude of the Earth globe in different image sequences. On the right-side of this figure, ‘*V=*’ represents the velocity (ground truth; unit: degree/frame) of the rotating Earth globe; and (**b**) the comparison of the calculated rotation amplitude with the ground truth of the Earth globe’s rotation.

Figure 9a shows the test results of the animated Earth globe’s rotation at nine different velocities using the optical flow algorithm. Figure 9b graphs the average rotation velocity of the animated globe and the deviation with the ground truth. As the rotation amplitude increases, the error increases sharply.

**Figure 10:**
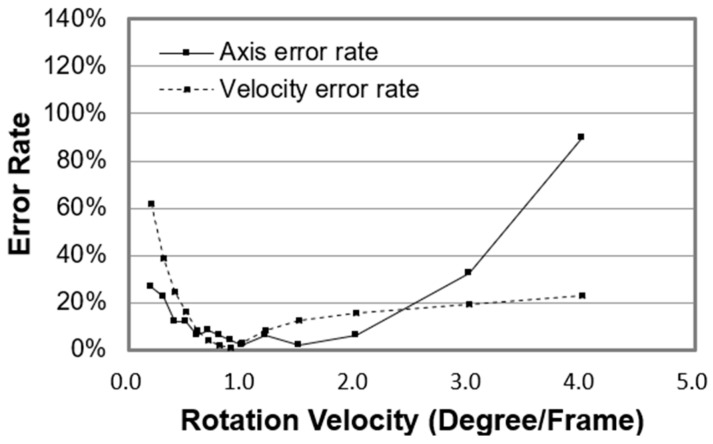
The error rates of the rotation axis and velocity.

Figure 10 shows the error rates of the rotation axis and velocity. The error rate of the rotation axis is defined as follows. If the angle between the calculated axis and the real axis is less than 10°, then the axis result is defined as correct; otherwise, it is incorrect. The error rate is the percentage of the incorrect results relative to the total results. As shown in Figure 10, if the error rate of the rotation axis is less than 20%, that of the rotation velocity will be less than 20% as well.

By comparing the testing results of Earth globe database and Backer’s template, we found that the image quality significantly affects the optical flow algorithm. The noise and the transparent member will sharply reduce the algorithm’s accuracy and the effective recognition range of the sphere’s rotation amplitude. In order to minimize the deviation between the measured data and real value, we applied this algorithm when the rotation velocity is between 0–4° per frame. If the sampling rate of the camera is 32 fps, the upper limit of the rotation velocity of the cell will be 134 rad per min (rpm). Then we used this method to test the rotation velocity of the Melan-A cells.

**Figure 11:**
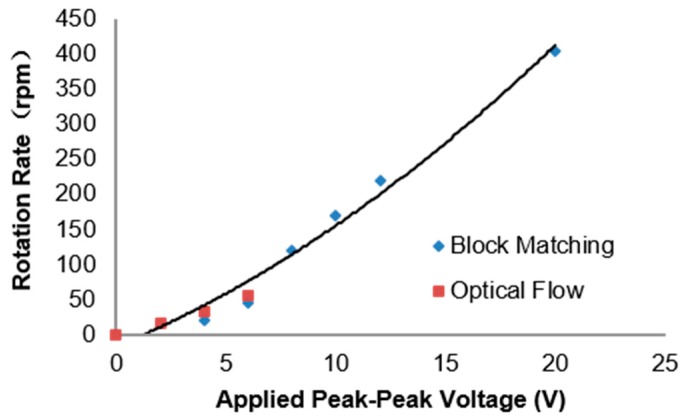
Calculated rotation velocity of the Melan-A cell in a 0.2 M sucrose solution versus the applied voltage from 0 Vpp to 20 Vpp at 40 kHz using different methods.

Figure 11 shows the rotation velocity calculated using the optical flow algorithm, which coincides with the results we got before using another method [14]. Since the sampling rate in the experimental video is 32 fps, we just measured the rotation velocity of these cells under the upper limit of 134 rpm. However, this limit can be lifted by increasing the sampling rate. As compensation, the instantaneous velocity and the rotation axis can be calculated.

**Figure 12:**
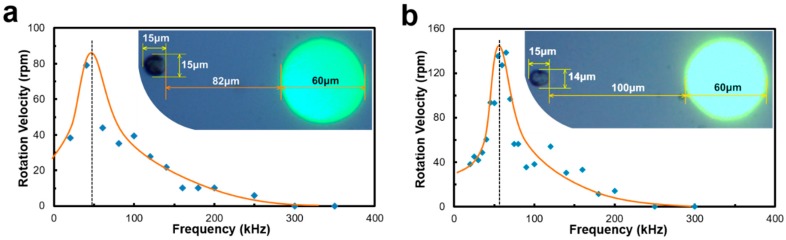
Rotation velocity of Raji cells versus applied frequencies. (**a**) The cell close to the electrode rotates slightly slower than (**b**) the cell far away from the electrode.

This algorithm was also used to measure the rotation velocity of Raji cells when different frequencies of the AC bias are applied to the OEK chip. Figure 12 shows two cells located in different places rotating around the light electrode. Compared to the cell in Figure 12b, the cell in Figure 12a is closer to the electrode, but its rotation speed is slightly lower, which may be caused by the difference of the cell membrane’s capacitance and conductance. This type of frequency-based cell rotation velocity spectrum may be a good indicator of cell types or cellular biophysical states. Through their specific rotation spectra, we expect to be able to identify cellular living states and drug responses.

## 4. Discussion and Conclusions

The accuracy of the calculated rotation velocity using the algorithm discussed in this paper depends heavily on two assumptions. The first assumption is that the texture feature of the cell is located on the surface and not intracellularly. The second assumption is that the cell is in a spherical shape. This second assumption provides an important theoretical precondition in the process of velocity feature recognition by mapping the 2D optical flow data to a 3D sphere model using orthographic spherical projection. Due to the sphere model’s isotropy this assumption makes the mapping process possible and simple. The anisotropy of other types of 3D models will make it tremendously difficult to determine the original orientation from where the target begins to rotate. In other words, this mapping process can only be accomplished based on 3D spherical models if there is no additional information indicating the orientation. However, perfect spherical shapes are hard to find in the real world, even for suspended cancer cells. Thus, this assumption is prone to bring an error to the calculation of the target’s rotation velocity. However, as the e algorithm is built upon operations on image pixels, at least thousands of pixels are required to determine the rotation axis and velocity together. Therefore, if the rotation target is in a shape similar to a sphere, or even an ellipse, this algorithm can also work well by using rotation data from major pixels and neglecting that from singular ones. In our experiments, the cells show very similar profiles when they rotate in the OEK chip at different angles. Therefore, this algorithm provides an appropriate means to calculate the rotation velocity and axis of cells.

The calculated rotation velocity result of the animated template is more accurate than that of the cell image sequence. There are three main reasons. (1) The experimentally acquired cell images have too much optical noise that cannot be rejected or filtered, but animated images do not have this problem. (2) The optical flow algorithm is too sensitive to the texture of the images. If the texture of the image sequence is very simple and does not vary sharply, the algorithm will produce a more accurate result. Conversely, if the texture is very complex and has large variations, there will be more errors in the result. (3) The size of the test images determines the computational time and affects the accuracy in identifying the rotation axis and the velocity.

In summary, we present in this paper a method to determine the cell rotation velocity in homogeneous AC electric fields based on an optical flow algorithm. The details of the algorithm and the testing results of two kinds of animated templates and two real cell rotation sequences were presented. The results demonstrate that this method is capable of performing the determination on the image sequence of single cells rotating around an unknown axis.
